# Unraveling the Genetic Etiology of Adult Antisocial Behavior: A Genome-Wide Association Study

**DOI:** 10.1371/journal.pone.0045086

**Published:** 2012-10-15

**Authors:** Jorim J. Tielbeek, Sarah E. Medland, Beben Benyamin, Enda M. Byrne, Andrew C. Heath, Pamela A. F. Madden, Nicholas G. Martin, Naomi R. Wray, Karin J. H. Verweij

**Affiliations:** 1 Genetic Epidemiology, Molecular Epidemiology, and Queensland Statistical Genetics Laboratories, Queensland Institute of Medical Research, Brisbane, Queensland, Australia; 2 Department of Psychiatry, Washington University School of Medicine, St. Louis, Missouri, United States of America; 3 School of Psychology, University of Queensland, Brisbane, Queensland, Australia; University of Iowa Hospitals & Clinics, United States of America

## Abstract

Crime poses a major burden for society. The heterogeneous nature of criminal behavior makes it difficult to unravel its causes. Relatively little research has been conducted on the genetic influences of criminal behavior. The few twin and adoption studies that have been undertaken suggest that about half of the variance in antisocial behavior can be explained by genetic factors. In order to identify the specific common genetic variants underlying this behavior, we conduct the first genome-wide association study (GWAS) on adult antisocial behavior. Our sample comprised a community sample of 4816 individuals who had completed a self-report questionnaire. No genetic polymorphisms reached genome-wide significance for association with adult antisocial behavior. In addition, none of the traditional candidate genes can be confirmed in our study. While not genome-wide significant, the gene with the strongest association (p-value = 8.7×10^−5^) was DYRK1A, a gene previously related to abnormal brain development and mental retardation. Future studies should use larger, more homogeneous samples to disentangle the etiology of antisocial behavior. Biosocial criminological research allows a more empirically grounded understanding of criminal behavior, which could ultimately inform and improve current treatment strategies.

## Introduction

Historically, the explanation of crime has shifted from a devil-based interpretation in medieval times into a more scientific interpretation, that is theory driven and multidisciplinary. In spite of the multidisciplinary approach of criminology, the last few decades have seen an almost purely environmental approach [1]. Despite the tremendous progress in molecular and behavioral genetics, modern biological approaches have been neglected by most criminological scholars to date. Nonetheless, biological insights seem indispensable in unraveling the etiology of criminal behavior and their incorporation into the explanation of crime should increase the explanatory power of criminology [2]. By elucidating genetic influences on antisocial behavior, a more sophisticated understanding of how the genetic liability of an individual ultimately leads to antisocial behavior can be achieved. Moreover, biological research may reveal the key elements that play a role in the interaction between certain environmental factors and genetic predisposition which would force criminology to expand its theories concerning the underlying biological underpinnings of criminal behavior [1].

It is known that crime related constructs such as conduct disorder [3], aggressive behavior [4], [5], rule-breaking behavior [6] and antisocial behavior [7] are substantially familial and likely heritable. However, few studies have tried to identify the specific genetic variants underlying this heritability. The present study therefore aims to contribute to biosocial criminology by conducting a genome wide association study on antisocial behavior. Previously, Dick et al. (2011) performed a genome-wide association study on conduct disorder, an antisocial syndrome that occurs in childhood and adolescence [8]. We performed the first GWAS on *adult* antisocial behavior.

### Adult antisocial behavior (AAB)

In the present study, we performed a genome-wide association test on a combined dataset, composed of phenotypic data from two cohorts. Adult antisocial behavior was measured by a diagnostic antisocial personality disorder (ASPD) and a non-diagnostic adult antisocial behavior questionnaire. Antisocial personality disorder (ASPD) is a mental health condition defined by the American Psychological Association (APA) as a disorder characterized by “…a pervasive pattern of disregard for, and violation of, the rights of others that begins in childhood or early adolescence and continues into adulthood” [9]. This definition emphatically includes an early start of maladaptive behavior and demands that the behavior is persistent. Evidence of conduct disorder with onset before the age of 15, is therefore stated as an essential condition for the diagnoses of ASPD. The prevalence of ASPD is higher in males (3%) than in females (1%) and shows a high co-morbidity with other psychiatric syndromes [10].

Research has shown that individual differences in antisocial behavior are due to both genetic and environmental influences [11], [12]. Ferguson et al. (2010) showed in a meta-analytic review of behavioral genetic studies, that genetic factors explain 56% of the variance in antisocial personality and behavior, while the remainder of the variance could be explained by unique environmental factors [13]. Moreover, a recent study by Tuvblad et al. (2011) suggested that the development of persistent antisocial behavior was primarily influenced by genetic factors, explaining 67% of the total variance [14]. These studies have highlighted the genetic propensity for displaying antisocial behavior. Candidate gene studies, looking at the association between specific genetic variants and a trait, have identified a number of genetic polymorphisms, such as dopaminergic (DAT1, DRD2, DRD4), serotonergic (5-HTTLPR) and enzymatic degradation (COMT, MAOA) genes related to a number of antisocial phenotypes [1]. Monoamine oxidase A (MAOA), for example, an enzyme that breaks down several monoamine neurotransmitters, has been associated with multiple antisocial phenotypes such as serious physical violence and gang membership [15]. Likewise, low serotonin concentrations (due to the short allele of 5-HTTLPR) have been linked to antisocial and violent behavior [16], [17]. However, candidate studies focusing on the genetic etiology of antisocial phenotypes have generally failed to replicate these genes, a phenomenon observed in genetic studies of other complex traits. For example, Verweij et al (2011), Bosker et al (2010), and Chabris et al (2011) were unable to replicate most of the candidate gene associations for cannabis use, depression, and intelligence [18]–[20]. Publication bias of candidate gene studies is one likely explanation [21].

Here, we use a hypothesis-free approach by scanning the entire genome to identify novel loci, rather than focusing on small candidate areas only. A previous study using a similar approach focused on the genetic variants underlying conduct disorder (CD), a childhood disorder that often precedes adult antisocial behavior. Dick et al. (2005) found four genome-wide significant (p<5*10^−8^) markers, two of which were located in a tumor necrosis factor-related gene (C1QTNF7) [8]. The authors state that it remains unclear whether this gene has a biologically relevant role in CD. To date, no genome-wide association study has been conducted on ASPD or any other adult antisocial phenotype. Therefore, we conducted the first GWAS in a large Australian sample of twins and their families to identify common genetic variants underlying variation in adult antisocial behavior.

## Methods

### 2.1 participants

A large community sample of twin pairs born between 1964 and 1971 were registered with the Australian Twin Registry (ATR) in 1980–1982 in response to media appeals and systematic approaches through the school system. The present study makes use of ATR participants, drawn from two studies that examined the role of genetic and social factors in drinking habits and co-morbid psychopathology, including antisocial behavior.

Data for the first study were collected between 1996 and 2000, by a telephone psychiatric interview containing lifetime assessments of several psychiatric disorders including adult antisocial behavior. This study cohort includes 1649 (43% male) participants, age range 24–41 (M = 31.2, SD = 3.5) and makes use of a non-diagnostic construct to measure adult antisocial behavior. Subjects in the second cohort were drawn from a series of studies as part of a Tobacco and Alcohol project, of which data were collected between 1981 and 2000. Study cohort 2 includes 3167 (41% male) individuals, who were aged between 18 and 81 (M = 47.6 years, SD = 9.5), and utilizes a diagnostic measure of antisocial personality disorder as its construct. Phenotypic and genotypic data collection was approved by the Queensland Institute of Medical Research (QIMR) Ethics Committee and informed consent was obtained from all participants. Phenotypic data on antisocial behavior were collected retrospectively using a semi-structured interview, administered by telephone. The total sample comprised of all the individuals for whom we had both genotypic and phenotypic data. Yielding a final study sample comprised of 4816 individuals from 2227 independent families.

### 2.2 Measurement

Adult antisocial behavior was determined from either a diagnostic assessment of ASPD (study 2) or a non-diagnostic measure of antisocial behavior (study 1). Participants in study 2 completed the Semi-Structured Assessment for the Genetics of Alcoholism [22], which includes a diagnostic assessment of antisocial personality disorder based on the criteria in the *Diagnostic and Statistical Manual of Mental Disorders* (4th ed. [*DSM–IV*]; [23]. The Tobacco and Alcohol project questionnaire yields scores on seven empirically derived syndrome scales, composed of 32 items that assessed antisocial behavior after the participant's 15^th^ birthday. Items include ‘Since age 15, have you been in physical fights?’ and ‘Have you often driven when you were high or drowsy on alcohol or drugs?’. Items were scored on a dichotomous scale (0 = no, 1 = yes). Responses were summed and clustered into the seven syndrome scales stated in the *DSM–IV* (such as deceitfulness, irresponsibility and aggressiveness). Case status was defined by the endorsement of three or more of the seven DSM–IV ASPD criteria as displayed under Criterion A in the statistical manual. Although we refer to this phenotype as ASPD case status throughout this article, full diagnostic criteria were not applied since Criterion D was not considered (the occurrence of antisocial behavior is not exclusively during the course of schizophrenia or a manic episode) in defining cases. Controls were specified as those who endorsed fewer than three symptoms for DSM-IV ASPD. In total, 122 subjects met these criteria for ASPD case status, while the control group consisted of 3045 individuals.

The non-diagnostic construct obtained from study 1, utilizes seven items related to antisocial behavior that also specifically address unlawful behavior, such as ‘Have you ever been arrested for anything?’ and ‘Have you ever spent time in jail?’. In this study, only those individuals who endorsed at least one of the DSM–IV criteria for conduct disorder were inquired about antisocial behavior. Case status was defined by the endorsement of three or more items, while controls were specified as those who endorsed fewer than three symptoms on antisocial behavior. In this study cohort, 176 subjects met criteria for case status, while the control group consisted of 1473 individuals.

For individuals who were present in both samples (n = 60) we retained the diagnostic criteria from Study 2. Missing items were replaced by the item sample mean and individuals with missing values on more than 25% of the items were removed from the dataset. The combined sample from the two studies comprised 298 cases and 4518 controls; the mean age of the cases was 33.3 years (SD = 8.9; range 18–74 years), while the mean age of the controls was 34.6 years (SD = 9.1; range 18–77 years).

### 2.3 Genotyping, quality control and imputation procedures

DNA samples were submitted for genotyping under a number of primary projects using different Illumina SNP platforms (Human610-Quad, HumanCNV370-Quadv3 and Human 317K). Standard quality control (QC) filters were applied to the genotyping in the different platforms. QC included checks for ancestry outliers, Mendelian errors, Hardy Weinberg Equilibrium, and Minor Allele Frequency (MAF) and was conducted separately for each of the projects. Thereafter, the combined dataset was screened for missingness within individuals, pedigree and sex errors, and Mendelian errors. Full details of the initial QC procedures for the Illumina and Affymetrix data can be found elsewhere [24]. Imputation to the European reference dataset (HapMap 1+2, Release 22 Build 36) was undertaken by means of MACH [25] using a set of Single Nucleotide Polymorphisms (SNPSs) common across all genotyping platforms. SNPs characterized by either a low minor allele frequency (MAF<.01) or a low imputation quality score (R^2^<0.3) were removed. Monozygotic twins that were not genotyped were assigned their co-twin's genotype. The final dataset included ∼2.4 million imputed autosomal SNPs and 13,783 genotyped X-chromosomal SNPs available for association analysis.

### 2.4 Statistical analyses

Prior to the GWAS analyses, we tested for sex and age effects in our sample in a linear regression model with binary adult antisocial behavior as the dependent variable. We conducted genome-wide association analyses in three study designs using imputation dosage genotypes: 1) combined studies, logistic regression on case-control status with sex, age and study as covariates 2) combined studies, linear regression on symptom count, same covariates as 1), 3) repeated analyses 1 and 2 for the two studies separately with age and sex as covariates. This allowed us to determine consistency among the associations across the studies. Given our family based sample, Merlin offline [26] was used since it accounts for family relationships including MZ twins. Minx (as implemented in Merlin) was used to perform association analyses on the X-chromosome. Ancestry principal components were not significantly associated with the phenotypes and were not included as covariates.

#### Gene-based test and pathway analysis

We tested for association at the level of genes using the versatile gene-based test for genome-wide association studies (VEGAS) [27]. While accounting for linkage disequilibrium (LD) and number of SNPs per gene, VEGAS aims to identify genes that show a higher signal of association than expected by chance, by considering all the p-values of all SNPs within genes (including ±50 kb from the 5′ and 3′ UTR). The gene-based association test was undertaken for 17,707 autosomal genes, we considered a p-value below α = 2.8×10^−6^ (0.05/17,707) to be significant. Since the MAOA gene is located on the X chromosome and sex chromosomes are not taken into account in VEGAS, we specifically checked all the SNPs in the MAOA gene that were covered in our dataset, to test if we could replicate the previously reported association in this gene.

A pathway analysis was carried out to determine which potential biological pathways could play a role in antisocial behavior. Pathway analysis was performed in the Ingenuity Pathway analysis program (Ingenuity Systems, release IPA6.0) using genes with a p-value below 0.01. Based on scientific literature, the Ingenuity database contains large amounts of up-to-date information concerning the localization, structure and biological functions of proteins and their interaction. Results were corrected for multiple testing using the Benjamini-Hochberg multiple testing correction as implemented in Ingenuity.

An approximate power calculation [28] indicates that the combined sample provided 50%, 72% and 87% power to detect a genetic variant (with a minor allele frequency of 0.25) with a relative risk of 1.4, 1.5 and 1.6, respectively.

#### Genome-wide Complex Trait Analysis

Furthermore, we performed a Genome-wide Complex Trait Analysis (GCTA) to estimate the proportion of the heritability of liability to adult antisocial behavior that can be explained by testing the SNPs on the GWAS chips simultaneously [29], [30]. One individual per family was selected for the analysis. We used only genotyped SNPs. To reduce the potential for bias, SNPs that had a Hardy-Weinberg p-value<10^−3^, had >5% missingness in all samples, or showed evidence of differential missingness between cases and controls (p<0.01), were removed. In this way only good quality SNPs genotyped across all genotyping platforms were retained. A total of 278.570 SNPs remained after quality control. A stringent cut-off of 0.025 was used to remove pairs of individuals that show evidence of cryptic relatedness. The final sample comprised 160 cases and 2012 controls. Analysis was performed using the GCTA software and all 22 autosomes were fitted in the model simultaneously. The prevalence estimate was 0.035% as estimated in the phenotypic sample.

## Results

Table 1 provides the means and standard deviations for antisocial behavior of both symptom count and case status derived from the two questionnaires. Consistent with findings in the literature, males had a significantly higher mean score than females on antisocial behavior (*p*<.001). Similarly, an age (of measurement) effect on the mean score was found. The mean score on antisocial behavior decreased as a function of age in our sample (*p*<.001). In order to overcome potential bias, we therefore adjusted for age and sex effects by including these variables as covariates in the association analyses. Moreover, because we used multiple study designs to operationalize adult antisocial behavior, study was also used as a covariate in the combined GWAS.

**Table 1:** Descriptive statistics for antisocial personality disorder (ASPD) and antisocial behavior (ASB).

	Males		Females	
Cohort	Cases	Controls	Cases	Controls
Study 1	129	585	47	888
Study 2	103	1189	19	1856
Combined	232	1774	66	2744

**Figure 1:**
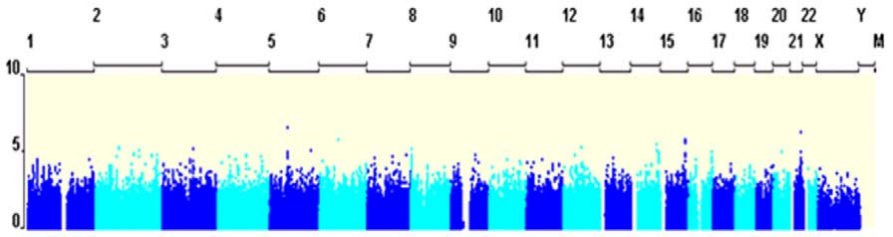
Manhattan plot showing the GWAS results of the combined study design for adult antisocial behavior. X-axis represents the chromosomal location for each SNP, and y-axis the −log10 p-value of the association signal.

**Figure 2:**
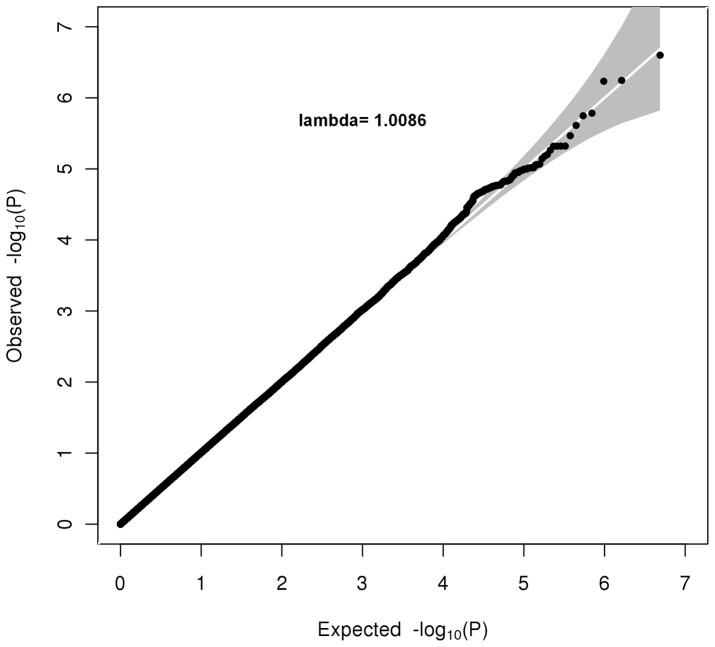
Quantile-Quantile plot showing the association between the observed and expected −log10 p-values. The grey shade area represents the 95% confidence interval. The plot shows the results of the combined study design.

The results of the association analyses on case status are summarized in Figure 1, and 2, and Table S1 that show the Manhattan plot, Quantile-Quantile (Q-Q) plots and the SNPs most associated with ASPD, respectively. The Manhattan plot in Figure 1 provides a graphical presentation of the association analyses in the combined study design. The strongest associations were located on chromosomes 5,14,15 and 21. However, none of these associations were genome-wide significant (*p*<5.0×10^−8^). Likewise, no SNPs reached genome-wide significance in the association analysis on symptom count of adult antisocial behavior. The genetic power calculation indicates that individual common genetic variants with a relative risk of ∼1.5 or greater do not contribute to individual differences in adult antisocial behavior. Figure 2 shows the Q-Q plots for each of the study designs, allowing inspection of systematic bias and population stratification by comparing the distribution of observed p-values with their expected distribution. The Q-Q plot lambda values are close to 1, indicating that the residual population stratification effect is minimal [31].

Table S1 lists the top 50 genetic markers showing the strongest association with our phenotype. The top SNPs explained less than 1% of the phenotypic variance, suggesting a highly polymorphic genetic architecture. Using these GWAS results we ran a gene-based association test aimed at finding evidence for association on a per gene basis. Table S2 displays the results of VEGAS and lists the 20 genes that showed the highest signal of association in our sample.

No genes met the criteria for genome-wide significance (p<2.8*10^−6^), but the most associated gene was Dual specificity tyrosine-phosphorylation-regulated kinase 1A (DYRK1A) gene (located at 21q22.13). Within the DYRK1A gene, 30 SNPs had a p-value below p<10^−5^ and there were an additional 96 SNPs that reached nominal significance (p<.05) in the gene, yielding converging evidence of association (see Figure S1). To see whether we could find independent evidence for involvement of this gene, we checked the associations in both study cohorts separately. The DYRK1A SNPs in the first study cohort yielded similar p-values as compared to the combined study design. In the second study cohort, no SNPs were significant at p<0.05.

We examined whether our top genes were more prevalent in any known biological or canonical pathway using genes associated with p-value<0.01. The pathway analyses showed that the top genes in our sample were not significantly more prevalent in any known pathway, although the biological pathway ‘Nervous System Development and Function’ showed the strongest association in our sample (p = .07, after correction for multiple testing).

We estimated the proportion of the heritability of liability to adult antisocial behavior explained by testing all the SNPs simultaneously using GCTA software. The estimated proportion of the phenotypic variance explained by the SNPs on the GWAS chips was 0.55 with a standard error of 0.41 and the estimate was not significantly different from zero (p = 0.07).

Finally, we checked whether the SNPs and genes that are previously associated with antisocial behavior could be replicated in our GWAS panel. Although several genetic polymorphisms related to antisocial phenotypes have been reported in the literature, follow-up studies attempting to replicate these findings reveal mixed results [32]. A list of candidate genes for antisocial phenotypes was gathered from published genetic association studies and gene expression studies. Table S3 contains an overview of the candidate genes that have been previously associated with antisocial phenotypes [33], displayed with their corresponding p-values as derived from our sample. Results indicate that none of the candidate genes reached nominal significance in our gene-based analyses, implicating that in contrast with these previous studies, we did not find evidence in our sample for involvement of these polymorphisms in adult antisocial behavior. Likewise, the genome-wide significant SNPs reported by Dick et al. (2011) did not reach nominal significance (*p*<0.05) in our sample. The MAOA gene is considered one of the most important candidate genes for antisocial phenotypes [33]–[39]. Since VEGAS does not take into account the X chromosome in its analyses, we tested all the SNPs across the MAOA gene that were covered by our GWAS panel. None of the seven MAOA SNPs yielded p-values below α = 0.05, implying no evidence for association of the MAOA gene in our sample (see Table S4).

## Discussion

Notwithstanding the enormous potential biology could offer criminology, there is still a relative paucity of biological research in the explanation of crime. The present study aims to contribute to biosocial criminology by performing the first genome-wide association analysis on adult antisocial behavior. Despite the substantial power to detect common genetic polymorphisms, no genome-wide significant SNPs were found. Nevertheless, the most associated gene DYRK1A (p = 8.70 * 10^−5^) reflected associations at three of our most associated SNPs (rs12106331, rs2835702 and rs2835771). The DYRK1A gene encodes for dual specificity tyrosine-phosphorylation-regulated kinase 1A, an enzyme that is thought to play a role in signaling pathway regulating cell proliferation and has been previously associated with synaptic plasticity and brain development [40], [41]. More specifically, DYRK1A is considered to be a strong candidate gene for mental retardation and is localized in the Down syndrome critical region of chromosome 21. Research has shown that early neuropsychological deficits might lead to poor cognitive functioning, emotional reactivity, and hyperactivity/impulsivity, all known as risk factors for antisocial behavior [42]. Terracciano et al. (2010) reported a nominal association (p = 3.0 * 10^−5^) of a SNP (rs2835731) within the DYRK1a gene with conscientiousness - a trait related to antisocial behavior [43]. Nevertheless, the associated SNP was not significant (p = 0.37) in our sample. We also tested for replication of the SNPs in the DYRK1a gene with conduct disorder in an American sample (N = 3963, 872 cases, 3091 controls, see Dick et al., 2010) [8]. None of the 99 tested SNPs reached significance after correcting for multiple testing, implying no evidence for replication.

Although several genetic polymorphisms related to antisocial phenotypes have been reported in the literature, follow-up studies attempting to replicate these findings have revealed mixed results [32], [44]. A list of candidate genes for antisocial phenotypes was gathered from published genetic association studies and gene expression studies. Results indicate that none of the candidate genes reached nominal significance in our sample, implicating that in contrast with these previous studies, we did not find evidence for involvement of these polymorphisms in adult antisocial behavior. However, since we did not test for gene environment interaction effects it is still possible that these genetic variants have relatively strong effects when linked with certain environmental factors. Previous studies have underscored the importance of taking into account the close interplay between genetic and environmental factors in the etiology of antisocial behavior. Caspi et al. (2002) showed for example that a functional polymorphism in the MAOA gene moderates the impact of childhood maltreatment on the development of antisocial behavior [45].

The discrepancy between the high heritability estimates in twin and adoption studies on the one hand, and the inability to identify genes involved in these behaviors on the other hand, has been often referred to as the problem of the ‘missing’ heritability [46]. While some genome-wide association studies have been successful in identifying common SNPs, the majority of genetic variants that contribute to disease susceptibility remain undiscovered [29]. Moreover, these associated genes typically explain only a small proportion (<1%) of the genetic variance underlying the trait. The power calculation shows that our sample is unable to detect common genetic variants of small effect sizes that contribute to the variance in antisocial behavior. Yang et al. (2010) showed that it is likely that the heritability is not ‘missing’, at least in part, but that the SNPs that tag certain genes have a very small effect individually and might therefore not be detected in the analyses [30], [47]. We estimated that the total proportion of phenotypic variance explained by genome-wide SNPs when considered together is 0.55, with a standard error of 0.41. The point estimate is non-significantly different from zero and larger sample sizes, enriched for cases, will be required to ensure sufficient power to accurately estimate the proportion of phenotypic variance in adult antisocial behavior explained by all the genome-wide SNPs. The application of this methodology to criminal behavior phenotypes is particularly relevant, a field in which a genetic contribution to the etiology remains contentious. Although the classical twin design for estimation of heritability is designed to separate out the common family environment effects from genetic effects in the familial relationship, some contamination with common environmental effects could remain [48]. The methods of Yang et al, estimate the contribution of genetic effects from such distantly relatives that contamination with family environmental effects is less likely.

Research has shown that it is likely that each gene associated with antisocial behavior affects many brain pathways (pleiotropy), while at the same time many genes affect each single brain pathway related to antisocial behavior (polygenicity) [1]. Hence, the genetic complexity of antisocial behavior makes it difficult to reveal causative genetic variants involved in this trait. Future research could therefore focus on functionally integrated brain networks, consisting of groups of genes, which are selected on the basis of their biological role. Functional gene group analyses are different from the pathway analysis conducted here, where we tested whether associated genetic variants are more prevalent in any known biological pathway. Instead, functional gene-group analysis tests whether the associated genes are more prevalent in any known functional gene-group (genes with a similar cellular function). As such, this analysis can deliver additional information to the field of criminology by complementing single SNP analysis [49]. Subsequently, genetic data combined with new biological techniques such as neuroimaging, could further explore the neurobiological underpinnings of criminal behavior by linking the genetic makeup of an individual to his neuroradiological features. Testing the hypothesis that there is a relationship between functional genetic networks, abnormalities in brain morphology and intra/inter-hemispheric connectivity related to antisocial phenotypes could be promising. Moreover, the neuroimaging data acquired can serve as an intermediate (endo-) phenotype and thus be used to form homogeneous groups of specific subtypes of antisocial behavior (such as aggression or conduct disorder), which improves biological interpretability as well as phenotypic differentiation under the assumption that different subtypes also have a different etiology [50].

Given the fact that criminology is in itself a highly multidisciplinary study, it is surprisingly that biological knowledge has been neglected by the majority of the criminological scholars the last few decades. There may be multiple reasons why criminologists have been cautious in applying biological theories to crime. The unpopularity of biosocial criminology is partly due to unfounded concerns regarding genetic determinism. Current biological approaches in criminology still suffer from the image of the Italian school of Cesare Lombroso in the nineteenth century [2]. In his most famous work ‘Criminal Man’ [51], Lombroso postulated that crime was caused by biological defects in inferior “atavistic” individuals who were “throwbacks” from an earlier evolutionary stage of human development. Although Lombroso published widely on the origins of delinquency, he is recognized and criticized most about his idea of physiognomy: the born criminal that could be distinguished by physical characteristics, such as large jaws and high cheekbones [52]. It is this reputation, a rather unsophisticated methodology used by early founders of biological theory that still puts biosocial criminology in a bad light. Nowadays the methodological tools have become one of the strengths of biology which is, as an exact discipline, characterized by empirical research and could therefore be of important value for criminology. Subsequently, the contemporary zeitgeist seems to be more receptive for further insights and the resistance against biology may gradually diminish [53].

Given the rise of modern biology in the explanation of crime, it is important to look ahead for the potential ethical implications that emanate with the emergence of neurobiological research. Crime is strongly related to our legal system and thereby impacts on typical legal concepts such as responsibility and free will, which explains why the use of biological techniques remains controversial [54]. Biosocial criminology urges a greater philosophical question whether an individual still has freedom to act if his behavior is biologically caused. Opponents argue that free will, as the foundation of our legal system, would be undermined if crime has genetic origins. It is clear that there are legal and ethical issues arising from behavioral genetics and neuroscience and these concerns should be taken into meticulous consideration [55]. In biosocial criminological circles it is widely acknowledged that there are ethical drawbacks to a strict biological approach and the large majority of these authors aim to have a biosocial perspective on crime rather than genetic determinism [1]. However, sometimes scientific findings are erroneously used by the uninitiated. Recently an Italian appeal court reduced the sentence of a murderer by one year, on the grounds of identifying the MAOA gene linked to violent behavior. It is exactly this type of events that shapes the fear of genetic research. Logically, behavioral geneticists from all over the world have challenged this ruling. Contemporary knowledge in genetics is surely not capable of predicting behavior at an individual level (as is clear from heritability estimates that are substantially less than one), but only in large population statistics [56].

Nevertheless, integrating biological research into the traditional sociological theories of crime, could be helpful in unraveling the complex etiology of criminal behavior. Ultimately, neuroscientific research could provide clues on which psychological or pharmacological interventions are suitable in improving the neurobiological pathways disrupted in antisocial individuals. To conclude, the study of crime has been eminently theoretical and lacks substantial empirical verification of those theories [57]. For these reasons, biological research could be of tremendous importance for criminology by incorporating empirical research into the traditional explanations of crime.

## Supporting Information

Figure S1 **Plot showing linkage disequilibrium and association of the SNPs in the DYRK1A region.**
(PDF)

Table S1 **50 strongest SNPs associated with adult antisocial behavior.**
(DOCX)

Table S2 **Top 20 genes showing strongest association with adult antisocial behavior.**
(DOCX)

Table S3 **Association results from the seven candidate genes previously found in antisocial phenotypes.**
(DOCX)

Table S4 Association results of the SNP p-values within the MAOA* gene in our sample.
(DOCX)
