# Supplementary material for: Unraveling the Genetic Etiology of Adult Antisocial Behavior: A Genome-Wide Association Study
Source: PLoS One. 2012 Oct 15;7(10):e45086. doi: 10.1371/journal.pone.0045086 (PMC3471931; doi:10.1371/journal.pone.0045086)
Supplement: Figure S1 — Plot showing linkage disequilibrium and association of the SNPs in the DYRK1A region. (PDF) [file pone.0045086.s001.pdf]

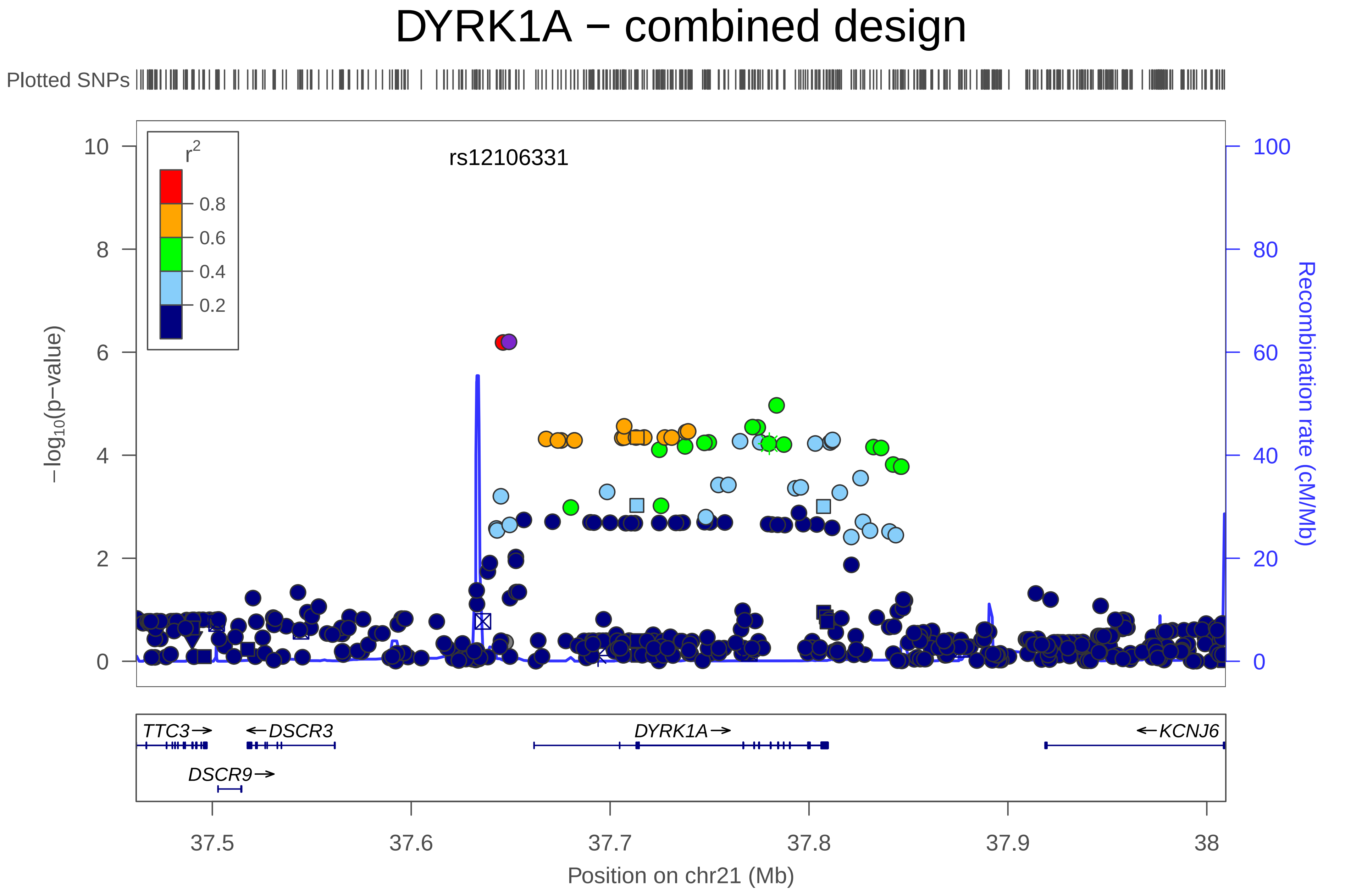

DYRK1A − combined design
Plotted SNPs
rs12106331
r2
0.8
0.6
0.4
0.2
−log10(p−value)
Recombination rate (cM/Mb)
0
2
4
6
8
10
0
20
40
60
80
100
TTC3→
←DSCR3
DYRK1A→
←KCNJ6
DSCR9→
37.5
37.6
37.7
37.8
37.9
38
Position on chr21 (Mb)

date:	Wed Jul 6 04:47:50 2011
build:	hg18
display range:	chr21:37461728−38009549 [37461728−38009549]
hilite range:	0 − 0 [0 − 0]
reference SNP:	rs12106331
number of SNPs plotted:	525
best p−value:	6.3E−7 [rs12106331]

annotation key

framestop	△
splice	△
nonsyn	▽
coding	□
utr	□
tfbscons	✳
mcs44placental	⊠
no annotation	○

Make more plots at http://csg.sph.umich.edu/locuszoom/
