# Supplementary material for: Unraveling the Genetic Etiology of Adult Antisocial Behavior: A Genome-Wide Association Study
Source: PLoS One. 2012 Oct 15;7(10):e45086. doi: 10.1371/journal.pone.0045086 (PMC3471931; doi:10.1371/journal.pone.0045086)
Supplement: Table S1 — 50 strongest SNPs associated with adult antisocial behavior. (DOCX) [file pone.0045086.s002.docx]

**Table S1. 50 strongest SNPs associated with adult antisocial behavior.**

| **Chr*** | **SNP** | **Base pair location** | **P-value** | **Minor Allele** | **MAF**** | **Beta** | **SE** | **Location** | **Imp/gen***** | **Closest gene** |
| --- | --- | --- | --- | --- | --- | --- | --- | --- | --- | --- |
| 5 | rs346425 | 62632000 | 2.51 × 10-7 | C | 0.486 | -0.026 | 0.005 | Upstream | imp |  |
| 21 | rs12106331 | 37649147 | 5.69 × 10-7 | A | 0.140 | -0.083 | 0.017 | Intergenic | imp | DYRK1A |
| 21 | rs2835702 | 37646181 | 5.84 × 10-7 | C | 0.140 | 0.036 | 0.007 | Intergenic | imp | DYRK1A |
| 6 | rs13202756 | 67856645 | 1.64 × 10-6 | A | 0.021 | -0.026 | 0.006 | Intergenic | imp | AL590874.1 |
| 15 | rs3829490 | 88574063 | 1.79 × 10-6 | C | 0.229 | -0.029 | 0.006 | Downstream | imp | CIB1 |
| 15 | rs3751656 | 88572708 | 2.44 × 10-6 | A | 0.231 | 0.028 | 0.006 | Synonymous coding | gen | SEMA4B |
| 14 | rs942746 | 90181831 | 3.41 × 10-6 | C | 0.378 | 0.024 | 0.005 | Intronic | imp | TTC7B |
| 12 | rs11176629 | 65887449 | 4.77 × 10-6 | G | 0.036 | 0.050 | 0.011 | Intergenic | imp |  |
| 12 | rs1657978 | 65889948 | 4.78 × 10-6 | C | 0.036 | 0.023 | 0.005 | Intergenic | imp |  |
| 12 | rs775313 | 65898396 | 4.79 × 10-6 | A | 0.036 | 0.066 | 0.015 | Intergenic | imp |  |
| 12 | rs2917849 | 65886130 | 4.79 × 10-6 | A | 0.036 | -0.066 | 0.015 | Intergenic | imp |  |
| 2 | rs7561888 | 86246279 | 5.46 × 10-6 | A | 0.022 | -0.083 | 0.018 | Non-coding region | imp | IMMT |
| 8 | rs6558776 | 3287571 | 6.29 × 10-6 | C | 0.016 | -0.107 | 0.024 | Intronic | imp | CSMD1 |
| 2 | rs17510310 | 86296719 | 6.63 × 10-6 | C | 0.021 | -0.070 | 0.016 | Downstream | imp | REEP1 |
| 3 | rs17722374 | 1.11E+08 | 7.15 × 10-6 | C | 0.055 | -0.023 | 0.005 | Intergenic | imp | RP11 |
| 14 | rs2277512 | 90183224 | 8.55 × 10-6 | A | 0.414 | -0.066 | 0.015 | Intronic | imp | TTC7B |
| 2 | rs3771684 | 1.6E+08 | 8.71 × 10-6 | C | 0.046 | -0.057 | 0.013 | Non-coding region | gen | BAZ2B |
| 5 | rs11956931 | 1.47E+08 | 8.79 × 10-6 | A | 0.014 | -0.036 | 0.007 | Non-coding region | imp | STK32A |
| 5 | rs443543 | 62600416 | 9.61 × 10-6 | A | 0.349 | 0.023 | 0.005 | Intergenic | imp |  |
| 14 | rs1385551 | 96444749 | 9.63 × 10-6 | C | 0.232 | -0.066 | 0.015 | Intergenic | imp | VRK1 |
| 5 | rs1676146 | 62596743 | 9.68 × 10-6 | C | 0.3489 | 0.078 | 0.018 | Intergenic | imp |  |
| 5 | rs458362 | 62596288 | 9.70 × 10-6 | A | 0.3489 | 0.023 | 0.005 | Intergenic | imp |  |
| 5 | rs460949 | 62595222 | 1.00 × 10-5 | C | 0.3492 | -0.023 | 0.005 | Intergenic | imp |  |
| 5 | rs460325 | 62595164 | 1.01 × 10-5 | A | 0.3492 | 0.023 | 0.005 | Intergenic | imp |  |
| 5 | rs462861 | 62594850 | 1.01 × 10-5 | G | 0.3492 | 0.023 | 0.005 | Intergenic | imp |  |
| 21 | rs2835771 | 37783703 | 1.05 × 10-5 | C | 0.263 | -0.025 | 0.006 | Intronic | imp | DYRK1A |
| 16 | rs11860152 | 84374825 | 1.06 × 10-5 | A | 0.0532 | -0.050 | 0.011 | Intronic;upstream | imp | COX4NB |
| 16 | rs11866179 | 84375919 | 1.07 × 10-5 | A | 0.0531 | -0.023 | 0.005 | Intronic | imp | COX4NB |
| 16 | rs896253 | 84377703 | 1.12 × 10-5 | A | 0.053 | 0.050 | 0.011 | Intronic | gen | COX4NB |
| 5 | rs373666 | 62594238 | 1.12 × 10-5 | C | 0.3499 | -0.023 | 0.005 | Intergenic | imp |  |
| 5 | rs401859 | 62594127 | 1.13 × 10-5 | A | 0.35 | 0.023 | 0.005 | Intergenic | imp |  |
| 20 | rs6119363 | 31340171 | 1.14 × 10-5 | C | 0.0118 | -0.154 | 0.036 | Intronic | imp | C20orf114 |
| 5 | rs444037 | 62606171 | 1.23 × 10-5 | A | 0.3681 | -0.023 | 0.005 | Intergenic | imp |  |
| 15 | rs9920862 | 88607144 | 1.24 × 10-5 | G | 0.2311 | -0.027 | 0.006 | Intronic | imp |  |
| 2 | rs9287528 | 1.4E+08 | 1.30 × 10-5 | G | 0.3508 | 0.023 | 0.005 | Intergenic | imp |  |
| 5 | rs349493 | 62592250 | 1.38 × 10-5 | C | 0.3465 | -0.023 | 0.005 | Intergenic | imp |  |
| 5 | rs409363 | 62612882 | 1.43 × 10-5 | C | 0.3606 | -0.022 | 0.005 | Intergenic | imp |  |
| 5 | rs424672 | 62612720 | 1.44 × 10-5 | A | 0.3606 | -0.022 | 0.005 | Intergenic | gen |  |
| 5 | rs369747 | 62606393 | 1.47 × 10-5 | A | 0.35 | -0.023 | 0.005 | Intergenic | imp |  |
| 5 | rs427421 | 62602801 | 1.48 × 10-5 | A | 0.3609 | -0.022 | 0.005 | Intergenic | imp |  |
| 5 | rs387996 | 62602687 | 1.49 × 10-5 | A | 0.3609 | -0.022 | 0.005 | Intergenic | imp |  |
| 5 | rs392246 | 62602498 | 1.49 × 10-5 | A | 0.3609 | -0.022 | 0.005 | Intergenic | imp |  |
| 5 | rs418226 | 62602292 | 1.49 × 10-5 | G | 0.361 | -0.022 | 0.005 | Intergenic | imp |  |
| 5 | rs434640 | 62597582 | 1.50 × 10-5 | A | 0.361 | -0.022 | 0.005 | Intergenic | imp |  |
| 5 | rs455575 | 62595583 | 1.54 × 10-5 | G | 0.3612 | 0.022 | 0.005 | Intergenic | imp |  |
| 2 | rs11901615 | 1.4E+08 | 1.58 × 10-5 | C | 0.3457 | -0.167 | 0.037 | Intergenic | gen |  |
| 4 | rs17008528 | 1.04E+08 | 1.62 × 10-5 | A | 0.027 | -0.086 | 0.019 | Non-coding region | imp |  |
| 5 | rs386661 | 62618589 | 1.69 × 10-5 | C | 0.3089 | -0.023 | 0.005 | Intergenic | imp |  |
| 5 | rs346431 | 62620271 | 1.69 × 10-5 | A | 0.3089 | -0.023 | 0.005 | Intergenic | imp |  |

*Chr=chromosome. ** MAF=Minor Allele Frequency. ***Imp= Imputed, Gen=Genotyped.
