# Supplementary material for: Unraveling the Genetic Etiology of Adult Antisocial Behavior: A Genome-Wide Association Study
Source: PLoS One. 2012 Oct 15;7(10):e45086. doi: 10.1371/journal.pone.0045086 (PMC3471931; doi:10.1371/journal.pone.0045086)
Supplement: Table S2 — Top 20 genes showing strongest association with adult antisocial behavior. (DOCX) [file pone.0045086.s003.docx]

**Table S2. Top 20 genes showing strongest association with adult antisocial behavior.**

| Chr* | Gene | N SNPs | Start position gene | Stop position gene | P-value gene | Best SNP | SNP p-value |
| --- | --- | --- | --- | --- | --- | --- | --- |
| 21 | DYRK1A | 235 | 37661728 | 37809549 | 8.70 x 10^-5^ | rs12106331 | 6.30 x 10^-7^ |
| 14 | VRK1 | 154 | 96333436 | 96417704 | 1.26 x 10^-4^ | rs1385551 | 1.02 x 10^-5^ |
| 2 | SMPD4 | 19 | 130625449 | 130656164 | 1.40 x 10^-4^ | rs13000721 | 7.70 x 10^-5^ |
| 2 | CCDC74B | 14 | 130613331 | 130619101 | 1.54 x 10^-4^ | rs13000721 | 7.70 x 10^-5^ |
| 2 | FAM128B | 19 | 130656172 | 130664770 | 1.54 x 10^-4^ | rs13000721 | 7.70 x 10^-5^ |
| 2 | TUBA3E | 15 | 130665787 | 130672504 | 2.09 x 10^-4^ | rs13000721 | 7.70 x 10^-5^ |
| 11 | ZBTB44 | 140 | 129601783 | 129689817 | 3.97 x 10^-4^ | rs4936094 | 7.55 x 10^-4^ |
| 2 | A26C1B | 18 | 130547577 | 130594610 | 5.23 x 10^-4^ | rs2084784 | 2.72 x 10^-4^ |
| 11 | ST14 | 132 | 129534891 | 129585467 | 5.24 x 10^-4^ | rs4936094 | 7.55 x 10^-4^ |
| 14 | CNIH | 65 | 53963396 | 53977898 | 5.50 x 10^-4^ | rs3204303 | 8.74 x 10^-5^ |
| 2 | A26C1B | 18 | 130547577 | 130594610 | 5.63 x 10^-4^ | rs2084784 | 2.72 x 10^-4^ |
| 14 | GMFB | 45 | 54010958 | 54025494 | 7.36 x 10^-4^ | rs17127582 | 1.92 x 10^-4^ |
| 15 | CPEB1 | 101 | 81009005 | 81113783 | 7.72 x 10^-4^ | rs3970696 | 9.14 x 10^-5^ |
| 1 | MAP1LC3C | 5 | 240225414 | 240228998 | 7.76 x 10^-4^ | rs10754748 | 3.56 x 10^-4^ |
| 1 | RBP7 | 21 | 9979860 | 9998665 | 8.51 x 10^-4^ | rs12140058 | 8.58 x 10^-4^ |
| 3 | LNP1 | 75 | 101602726 | 101657860 | 8.54 x 10^-4^ | rs4928050 | 3.47 x 10^-5^ |
| 1 | RBP7 | 21 | 9979860 | 9998665 | 9.14 x 10^-4^ | rs12140058 | 8.58 x 10^-4^ |
| 15 | SEMA4B | 124 | 88529155 | 88573896 | 1.01 x 10^-3^ | rs3829490 | 1.74 x 10^-6^ |
| 7 | CD36 | 178 | 80069439 | 80146529 | 1.04 x 10^-3^ | rs3211834 | 2.97 x 10^-4^ |

*Chr=chromosome.
