# Supplementary material for: Unraveling the Genetic Etiology of Adult Antisocial Behavior: A Genome-Wide Association Study
Source: PLoS One. 2012 Oct 15;7(10):e45086. doi: 10.1371/journal.pone.0045086 (PMC3471931; doi:10.1371/journal.pone.0045086)
Supplement: Table S3 — Association results from the seven candidate genes previously found in antisocial phenotypes. (DOCX) [file pone.0045086.s004.docx]

**Table S3. Association results from seven candidate genes previously found in antisocial phenotypes.**

| *Gene* | *Chromosome* | *Start*  *position* | *End*  *position* | *N tagged*  *SNPs* | *P-value* | *Rank* | *SNPs at*  p < *0.05/*  p < *0.01* | *% of gene*  *variants*  *covered* |
| --- | --- | --- | --- | --- | --- | --- | --- | --- |
| DAT1 | 16 | 74190498 | 74214655 | 85 | 0.75 | 12837/17707 | 0/0 | 95% |
| DRD2 | 11 | 112785526 | 112851211 | 197 | 0.89 | 15389/17707 | 0/0 | 100% |
| DRD4 | 11 | 627304 | 630703 | 46 | 0.61 | 10268/17707 | 0/0 | 93% |
| 5-HTTLPR | 17 | 25549031 | 25586841 | 85 | 0.76 | 13026/17707 | 3/0 | 97% |
| COMT | 22 | 18309308 | 18336530 | 132 | 0.30 | 5111/17707 | 0/0 | 98% |
| MAOA | - | - | - | - | - | - | - | - |
| C1QTNF7 | 4 | 14950709 | 15056364 | 256 | 0.49 | 8299/17707 | 6/0 | 88% |
