# Supplementary material for: Unraveling the Genetic Etiology of Adult Antisocial Behavior: A Genome-Wide Association Study
Source: PLoS One. 2012 Oct 15;7(10):e45086. doi: 10.1371/journal.pone.0045086 (PMC3471931; doi:10.1371/journal.pone.0045086)
Supplement: Table S4 — Association results of the SNP p-values within the MAOA* gene in our sample. (DOCX) [file pone.0045086.s005.docx]

**Table S4. Association results of the SNP p-values within the MAOA* gene in our sample.**

| SNP | P-value |
| --- | --- |
| rs1137070 | 0.5444 |
| rs12843268 | 0.9188 |
| rs1465108 | 0.3868 |
| rs1800464 | 0.576 |
| rs2072743 | 0.7771 |
| rs2235186 | 0.551 |
| rs6610845 | 0.6944 |
| rs909525 | 0.9331 |

*MAOA= Monoamine Oxidase A.
